# Primary total knee replacement for tibial plateau fractures in older patients: a systematic review of 197 patients

**DOI:** 10.1007/s00402-021-04150-1

**Published:** 2021-08-31

**Authors:** V. Tapper, A. Toom, K. Pamilo, T. Niinimäki, J. Nieminen, S. Nurmi, T. Kortekangas, J. Paloneva

**Affiliations:** 1 Central Finland Hospital, Keskussairaalantie 19, 40620 Jyväskylä, Finland; 2 Oulu University Hospital, Oulu, Finland; 3 Coxa Hospital for Joint Replacement, Tampere, Finland; 4 Tampere University Hospital, Tampere, Finland; 5 University of Eastern Finland, Kuopio, Finland

**Keywords:** Surgery, Knee, Tibial plateau fracture, Total knee replacement, Primary total knee replacement, Osteosynthesis

## Abstract

**Introduction:**

Tibial plateau fractures are typically treated with osteosynthesis. In older patients, osteosynthesis is associated with some complications, risk of post-traumatic osteoarthritis and long partial, or non-weight bearing during the recovery phase. To avoid these problems, primary total knee replacement (TKR) has become an increasingly common treatment option. The aim of this study was to evaluate all the relevant literature and summarize the current evidence-based knowledge on the treatment of tibial plateau fractures with primary TKR in older patients.

**Materials and methods:**

A systematic literature search of studies on total knee replacement (TKR) as primary treatment for acute traumatic tibial plateau fracture was conducted using OVID Medline, Scopus, and Cochrane databases from 1946 to 18 November 2019. We included all studies without restrictions regarding total knee replacement (TKR) as primary treatment for acute traumatic tibial plateau fracture.

**Results:**

Of the 640 reviewed articles, 16 studies with a total of 197 patients met the inclusion criteria. No controlled trials were available, and the overall quality of the literature was low. The results, using different clinical scoring systems, were good or fair. Four-year follow-up complication (6.1%) and revision (3.6%) rates after primary TKR appeared to be lower than after secondary TKR (complication rate 20–48%, revision rate 8–20%) but higher than after elective primary TKR.

**Conclusion:**

Based on low-quality evidence, TKR appears to be a useful treatment option for tibial plateau fractures in older patients. Controlled trials are mandatory to determine the relative superiority of these two options as primary treatment of tibial plateau fractures in older patients.

**Supplementary Information:**

The online version contains supplementary material available at 10.1007/s00402-021-04150-1.

## Introduction

For many years, arthroplasty has been the gold standard treatment for femoral neck fractures in older patients. More recently, arthroplasty has also become a significant primary treatment option for complex elbow and shoulder fractures [1, 2]. It has been assumed that peri-articular fractures around the knee can be similarly treated, especially in patients with osteoporosis and with fractures that are difficult to reconstruct or may lead to rapid progression of post-traumatic osteoarthritis.

Proximal tibia fractures are relatively common in the older population, but demanding to treat. Tibial plateau fractures, in turn, have been associated with higher mortality [3]. The incidence of tibial plateau fractures rises with age, and the majority of patients are women [4, 5]. Of all intra-articular proximal tibia fractures, 24% occur in older persons and account for 8% of all fractures in patients over age 65 years [6]. The incidence of tibial plateau fractures will increase in the future [6–8].

Conflicting results have been reported for open reduction and internal fixation (ORIF) of tibial plateau fractures in older people. Some studies have found a high risk of complications, such as infections, loss of reduction, malalignment, delayed union or nonunion, and risk for the development of post-traumatic osteoarthritis (OA) [6, 9–15]. However, many other studies have reported ORIF to have good outcomes in older people [8, 16–20].

In view of the above-mentioned complications, primary total knee replacement (TKR) is an under-reported treatment option. It could be a feasible first-line treatment, especially in fragile older patients [21–25]. Here we present a systematic review of all the relevant literature and summarize the current evidence-based knowledge on the treatment of tibial plateau fractures with primary TKR in older people.

## Materials and methods

We conducted a systematic search following the Preferred Reporting Items for Systematic Reviews and Meta-analyses (PRISMA) using a Prisma checklist. The review was registered with PROSPERO (CRD42020102352).

### Literature search

**Fig. 1:**
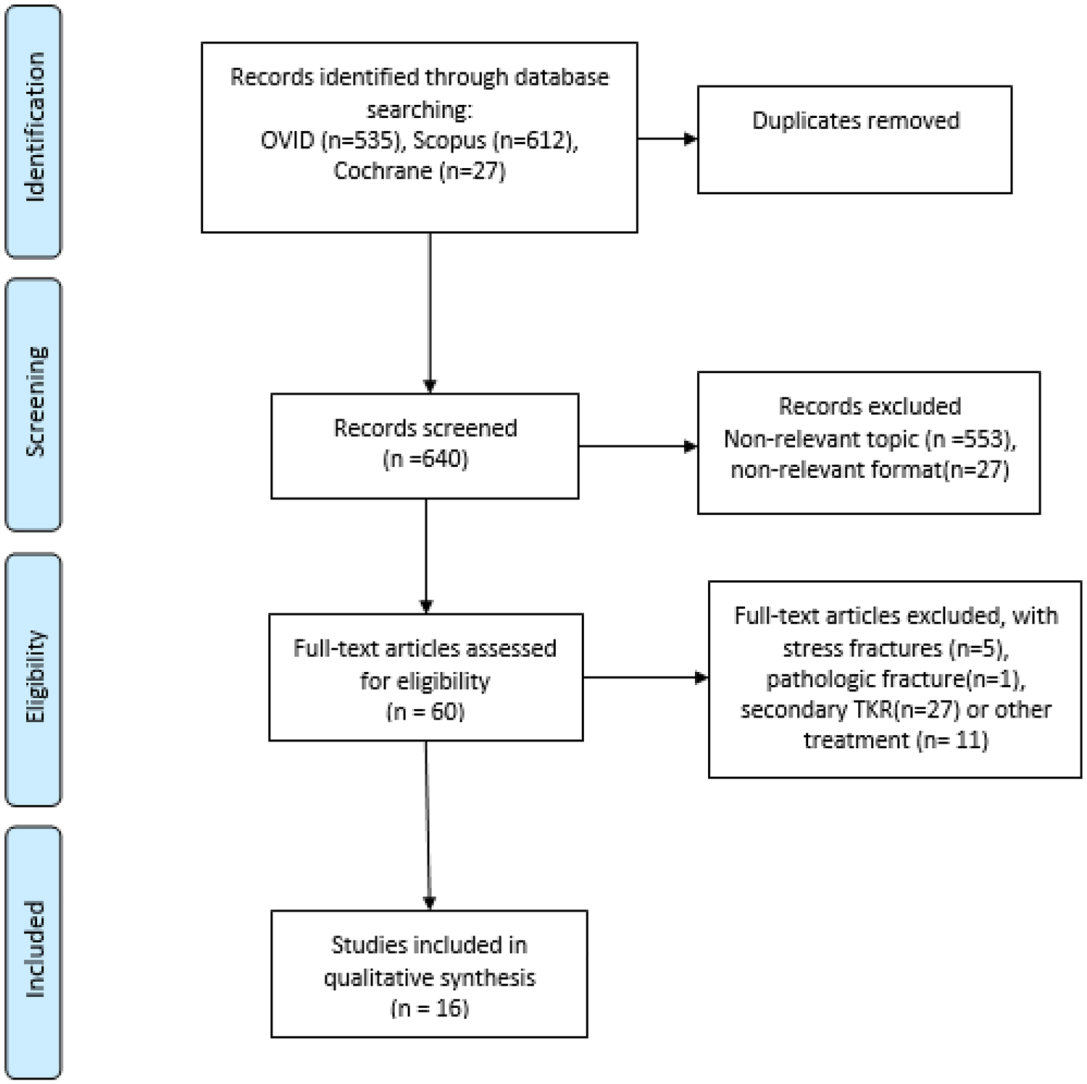
Flowchart of study selection

The literature search was conducted in three electronic databases (OVID Medline, Scopus, and Cochrane). The search covered the years 1946–2019, with the last search for 18.11.2019. The search strategy was developed by an experienced informatician, using the text words”knee joint replacement”, “knee TEP”, “total knee arthroplasty”, “knee arthroplasty” and “tibial head fractures”, “proximal tibia* fractur*”,”tibial plateau fracture*”, “proximal tibia* fracture”, “tibial plateau fracture”, “knee joint fractures”,” tibial fractures not femoral fractures”. The MESH terms are detailed in the electronic supplementary material. The study selection flowchart is presented in Fig. 1.

### Inclusion and exclusion criteria

All English, German, and French studies on TKR as primary treatment for acute tibial plateau fracture were included. The inclusion criteria for this review were based on the PICOS framework (see Table 1).

**Table 1:** PICOS

Population	Patients with acute traumatic tibial plateau fracture
Intervention	Primary total knee replacement
Comparison	Any other treatment
Outcome	Any outcome
Studies	All studies excluding conference abstracts, book chapters, reviews etc

Studies of osteosynthesis, external fixator, or any other treatment were excluded, as were studies on stress fractures, intra-operative fractures, pathologic fractures, and tibial fractures treated with secondary TKR. Conference abstracts, book chapters, and reviews were also excluded from our search.

### Data screening and extraction

The database search yielded 640 abstracts after removal of duplications. To exclude irrelevant records, conference abstracts, reviews, and expert opinions, two authors (AT, VT) performed title and abstract screening independently. The full-text selections from the remaining 55 articles were subsequently performed by the same two authors independently. Discrepancies were resolved through discussion.

Five of the selected studies concerned peri-articular knee fractures treated with primary TKR. Patients in these studies were excluded if cases with distal femoral fractures or extra-articular tibial fractures could not be separated from cases with tibial plateau fractures [24, 26–29]. Subject parameters were excluded where data were unreported or unclear.

### Outcome measures

Study and subject parameters were collected by the same author (VT). The collected parameters included year of publication, quality of evidence, length of follow-up, sex, age, complications, reoperations, fracture type, the type of prosthesis used, and surgical outcomes measured by the following clinical scoring systems: Oxford Knee Score (OKS; range 12–60 points) [30], Knee Society Score (KSS; range 0–200 points) [31], Tegner Activity Scale (Range 0–10) [32] and Parker Mobility Score(Range 0–9) [33]. In all the above, a higher score indicates better knee function. Clinical scores were recorded at the last follow-up visit. Four superficial infections, three hematomas and one other wound-related problem were excluded owing to insufficient documentation. Deep infections were defined as infections requiring surgical revision. All other complications (deep infection, intra-operative fracture, periprosthetic fracture, prosthetic loosening, deep vein thrombosis (DVT), knee stiffness requiring revision) reported in the original articles were recorded.

### Statistical analysis

A meta-analysis of case series was not possible owing to the diversity of outcomes measures, unclear data, and a low event rate. Comparison of main outcomes by fracture classification and by complications between the prosthetic models used were quantitatively analyzed with the SPSS data analysis program, and *p* value < 0.05 was considered statistically significant. Descriptive results of the included studies are expressed as absolute numbers with percentages, mean values, and standard deviations.

## Results

Sixteen series on 197 patients met the inclusion criteria. No prospective controlled trials were found. The included studies are presented in Tables 2 and 3.

**Table 2:** Studies included in the review

Publisher	Country	Year	Level of evidence	Patients	Mean follow-up time (months)
Kilian et al. [37]	Germany	2003	IV	2	2
Nau et al. [26]	Austria	2003	IV	3	Unclear data
Nourissat et al. [12]	France	2006	IV	4	42
Schwarz et al. [34]	Austria	2008	IV	9	16
Vermeire et al. [21]	Belgium	2010	IV	12	31
Malviya et al. [27]	UK	2011	IV	15	39
Kini et al. [38]	Singapore	2013	IV	6	Unclear data
Boureau et al. [24]	France	2015	III	11	31
Haufe et al. [25]	Germany	2016	IV	30	19
Shimizu et al. [16]	Japan	2016	IV	2	27
Sarzaeem et al. [23]	Iran	2017	III	30	48
Huang et al. [22]	China	2017	IV	6	36
Ebied et. al. [28]	Egypt	2018	IV	18	32
Abdelbadie et al. [36]	Egypt	2019	III	22	27
Tapper et al. [35]	Finland	2019	IV	22	19
Pasurka et al. [29]	Germany	2019	III	5	Unclear data

**Table 3:** Reported outcome of tibial plateau fractures in the included studies

Publisher	Patients	Mean follow-up time (months)	Complications	KSS knee score*	KSS function score*	Global KSS (Knee + function) score*	OKS*	Tegner score*	Parker score*	Flexion degrees*	Mean age (years)	Gender (female)
Haufe et al. [25]	30	19	Infection, i.op. fracture, loosening	81.1	74.5	155.6	–	–	–	–	86	–
Sarzaeem et al. [23]	30	48	0	90.7	69.6	160.3	–	2.5 – > 3.5	–	106	69.5	50%
Tapper et al. [35]	22	19	Infection, stiffness	–	–	160	27	–	–	109	78.4	57%
Abdelbadie et al. [36]	22	27	Periprosthetic fracture	83	84	167	–	–	–	115	67.1	64%
Ebied et. al. [28]	18	32	0	–	–	–	–	–	–	–	74.4	63%
Malviya et al. [27]	15	39	–	–	–	–	–	–	–	–	80	–
Vermeire et al. [21]	12	31	DVT, periprosthetic fracture	78	58	136	–	–	–	116	73	75%
Boureau et al. [24]	11	31	–	84	43	127	35,7	–	7.4 – > 5	–	79	–
Schwarz et al. [34]	9	16	2 infection	–	–	170	–	–	–	107	70	–
Kini et al. [38]	6	–	–	–	–	–	–	–	–	–	–	–
Huang et al. [22]	6	36	0	–	–	–	–	–	–	119	–	–
Pasurka et al. [29]	5	–	0	–	–	–	–	–	–	102	78.4	–
Nourissat et al. [12]	4	42	DVT	81	69	150	–	–	–	98	81.8	–
Nau et al. [26]	3	–	0	–	–	–	–	–	–	–	74.7	100%
Kilian et al. [37]	2	2	0	–	–	–	–	–	–	103	73.5	50%
Shimizu et al. [16]	2	27	–	–	–	–	–	–	–	–	67.6	37%

*KSS* Knee society score, *OKS* Oxford knee score, *DVT* Deep vein thrombosis, *–* unreported or unclear data

*Clinical scores were recorded at last follow-up visit at end of follow-up time

Mean patient age ranged between 68 and 86 (SD 6.3) years [16, 21–27, 29, 34–37].

Mean follow-up time ranged from 2 months to 4 years (mean 28 months, SD 13 months) [12, 16, 21–27, 34–37]. Studies using the AO classification identified seven A-type (1%), 70 B-type (65%) and 31 C-type (39%) tibia fractures in a total of 108 patients [21, 23, 25–27, 29, 34, 37]. The six series using Schatzker’s tibia fracture classification [12, 22, 24, 35, 36, 38] identified 28 II-type (39%), 15 III-type (21%), 14 IV-type (20%), 11 V-type (15%), and three VI-type (4%) fractures in 71 patients.

The OKS score was 29.5 and 35.7 points in 24 patients [24, 35]. Reported KSS scores ranged from 127 to 170 points (140 patients), function scores from 43 to 84 points and knee scores from 78 to 90.7 points (87 patients) [12, 21, 23–25, 34–36]. The mean global KSS score was 153 (SD 15) points. The mean KSS score was 150 (SD 43) points in patients with B-type fractures (N = 26) and 144 (SD 38) points in patients with a bicondylar C-type fracture (N = 14). In the remaining cases, fracture type was not reported. The difference between B and C-type fractures was not statistically significant (*p* = 0.709). Maximum flexion was 98–116 degrees in 101 patients [11, 19–21, 27, 33–37]. In the reviewed studies, mean maximum flexion was 108 (SD 7) degrees, in patients with B-type fractures (27 patients) 110 (SD 13) degrees, and in patients with bicondylar C-type fracture (14 patients) 109 (SD 15) degrees (p = 0.738 between groups). No statistically significant difference between fracture groups was found. In the remaining cases, fracture type was not reported. Complications were reported in 12 studies (infection, periprosthetic fracture, loosening, stiffness, thromboembolism) [12, 21–23, 25, 26, 28, 29, 34–37]. Ten complications (6.1%) and six (3.6%) revisions were reported in 163 patients during variable follow-up between studies (see Table 3). Despite conversion of the Schatzker classified fractures to the AO classification, the event rate was too low for further comparison between complications and fracture classification.

Prostheses were categorized by stability into three types: hinge, total stabilized, and surfacing prostheses (cruciate retaining or posterior stabilized). The prosthesis used was reported in 144 (75%) patients [21, 23, 24, 27, 35–37]: 46 (32%) were hinged, 32 (22%) total stabilized, and 66 (46%) surfacing prostheses.

## Discussion

The overall quality of the literature was low. Based on the limited evidence in the reviewed studies, TKR appears to be a useful treatment option for tibial plateau fractures in older patients with acceptable complication risk. However, according to the results of this review study, the choice between ORIF and TKR cannot be made based on the currently available literature.

Many different scoring systems were used to report the outcome of primary TKR for acute tibial plateau fractures in the reviewed studies. The scores showed a good to fair outcome in the majority of cases after a mean follow-up of 28 months. The complication rate after primary TKR appears to be lower than after secondary TKR but higher than after elective primary TKR, and the revision rate followed the same pattern. [9, 39, 40] These results suggest that primary TKR is a potentially useful choice in future cases, as it would enable minimal use of secondary TKR with its higher complication rate.

Unfortunately, the distribution of fracture types and the prosthetic models used were reported in relation to outcome or complications in only seven of the reviewed studies. Due to the variety of outcome measures, unclear data, and a low event rate, comparison of main outcomes by fracture classification was only possible with the maximum flexion and KSS scores. The difference found was not statistically significant. For the same reasons, comparison of the results and of complications between the prosthetic models used was also not possible.

Furthermore, patient morbidity was reported only in one study (ASA classification) and thus was not included in this review [24]. While no life-threatening complications related to primary TKR operation were reported in the reviewed studies, more studies comparing mortality between primary TKR and ORIF treatment are needed.

Only two studies included a (small) control group treated with ORIF. Abdelbadie et al. reported better range of motion and functional scores in TKR than ORIF patients. Pasurka et al. reported that patients with primary TKR achieved independent mobility earlier than patients treated with ORIF [29]. In both studies, complication and reoperation rates were also lower in the primary TKR group [29, 36].

A major weakness of the data was that only two studies compared the postoperative outcome to the pre-fracture situation [23, 24]. Thus, achieving the main goal, both functional rehabilitation and maintenance of autonomy after TKR, remains controversial [23, 24]

## ORIF

With ORIF, the goals are to achieve anatomical reduction, joint reconstruction, and high stability to allow early weight-bearing. However, to acceptably reduce the joint surface, achieve knee stability and restore the mechanical axis can be challenging due to possible complexity of the fracture or inferior bone quality. ORIF has shown good results in some studies, with low rates (0–9.5%) of complications in older patients [8, 9, 13, 16, 17, 19]. However, some problems seem to be associated specifically with tibial plateau fractures treated using ORIF. Postoperative complications of ORIF include deep infection (16%), malalignment, loss of reduction (30–79%), and delayed union or nonunion [6, 10, 18, 41–44]. Moreover, the risk of complications has been reported to rise with age [10, 17, 18, 41].

The postoperative management of ORIF may be challenging. In most cases, to achieve fracture union in older patients with poor bone quality, a relatively long period of non- or partial weight bearing is warranted. Such immobilization leads to loss of muscle strength, joint contractures, increased risk of venous thromboembolism, bed sores, and prolonged hospital stay [45–47].

Even if adequate reduction and stable fixation are achieved, 21–75% of patients with intra-articular knee fractures develop post-traumatic OA [13–15]. In comparison to the normal population, these patients are at a 5.3-fold risk for post-traumatic osteoarthritis [11]. However, the rates reported for secondary total knee arthroplasty are relatively low. End-stage osteoarthritis leading to secondary total knee arthroplasty typically develops in 4–7.5% of patients after a median of 3.7–4.6 years post-trauma, and the risk of end-stage osteoarthritis rises with age [11, 48, 49].

## TKR

Secondary arthroplasty may be considered in cases of failure or poor results after primary ORIF; however, it may be technically challenging owing to distorted anatomy, scarring and internal fixation materials [39, 50]. Moreover, achieving the correct ligamentous balance and patellar tracking, and restoring axial alignment are demanding [51]. The risk of revision surgery after secondary TKR varies from 8 to 20% in follow-ups lasting up to 11 years [9, 39, 40, 52] and the risk is 1.2–2.4 times higher than after primary OA [53, 54]. After 5 years, it seems that no significant differences in revision risk remained between these two groups [54]. Moreover, the complication rate of secondary TKR is as high as 24–48% in follow-ups lasting up to 6.2 years [9, 39, 52]. The infection risk is 2.9-fold higher in cases of post-traumatic arthritis compared to TKR performed due to primary osteoarthritis [55].

The majority of the reviewed studies concluded that primary TKR for tibial plateau fracture is a potential treatment option in well-selected patients. Primary TKR is probably a useful primary treatment option in older patients, especially in cases where (1) the patient is a likely candidate for TKR in the near future due to severe pre-existing OA of the knee, when even successful ORIF treatment of a fracture may result in a stiff and painful knee [56]; (2) the fracture is combined with marked bone loss/defects that are difficult to repair or reconstruct; and (3) patient compliance with partial weight-bearing is insufficient and thus ORIF would lead to immobilization.

The advantages of primary TKR are early mobilization, faster rehabilitation, and an assumed decrease in reoperations, achieved through avoiding complications such as malalignment, loss of reduction, and secondary osteoarthritis after ORIF. The weaknesses of primary TKR are the more demanding treatment required in cases of infection and the limited possibilities for revision in cases of complications due to a voluminous prosthesis. However, the implant should survive for the rest of the patient’s life, and hence is preferred for older patients.

## Limitations of the study

The quality of the reviewed literature was low (grade 3–4). Moreover, no studies included controlled trials: most were case reports with only a small number of patients.

## Conclusion

No conclusions on the relative superiority of TKR or ORIF as treatment for tibial plateau fractures can be drawn, owing to the low quality of the literature and lack of studies with control groups. To obtain more precise guidelines for such treatment, we need controlled studies that assess the functionality of TKR and ORIF in addition to fracture healing, patient satisfaction and health-related quality of life.

## Supplementary Information

Below is the link to the electronic supplementary material.

Supplementary file1 (DOCX 18 KB)
