# Supplementary material for: Primary total knee replacement for tibial plateau fractures in older patients: a systematic review of 197 patients
Source: Arch Orthop Trauma Surg. 2021 Aug 31;142(11):3257–64. doi: 10.1007/s00402-021-04150-1 (PMC9522836; doi:10.1007/s00402-021-04150-1)
Supplement: Supplementary file 1 — Supplementary file1 (DOCX 18 KB) [file 402_2021_4150_MOESM1_ESM.docx]

### **Supplementary data**

Database: Ovid MEDLINE(R) ALL / PubMed(R) <1946 to Present> Search Strategy:

--------------------------------------------------------------------------------

1 Tibial Fractures/ (14903)

2 Tibia/ (34033)

3 Knee Joint/ or Knee Injuries/ or Knee/ (75569)

4 2 or 3 (102925)

5 Fractures, Bone/ (62930)

6 Fracture Healing/ (12949)

7 (fracture* or schatzker*).mp. [mp=title, abstract, original title, name of substance word, subject heading word,

floating sub-heading word, keyword heading word, organism supplementary concept word, protocol supplementary concept word, rare disease supplementary concept word, unique identifier, synonyms] (296759)

8 5 or 6 or 7 (296759)

9 4 and 8 (10758)

10 1 or 9 (21795)

11 (tibia* adj3 fracture*).mp. [mp=title, abstract, original title, name of substance word, subject heading word,

floating sub-heading word, keyword heading word, organism supplementary concept word, protocol supplementary concept word, rare disease supplementary concept word, unique identifier, synonyms] (17756)

12 (knee* adj3 fracture*).mp. [mp=title, abstract, original title, name of substance word, subject heading word,

floating sub-heading word, keyword heading word, organism supplementary concept word, protocol supplementary concept word, rare disease supplementary concept word, unique identifier, synonyms] (1334)

13 (tibiofemoral* adj3 fracture*).mp. [mp=title, abstract, original title, name of substance word, subject heading

word, floating sub-heading word, keyword heading word, organism supplementary concept word, protocol supplementary concept word, rare disease supplementary concept word, unique identifier, synonyms] (4)

14 (tibia* adj5 Schatzker*).mp. [mp=title, abstract, original title, name of substance word, subject heading word,

floating sub-heading word, keyword heading word, organism supplementary concept word, protocol supplementary concept word, rare disease supplementary concept word, unique identifier, synonyms] (167)

15 (tibiofemoral* adj5 Schatzker*).mp. [mp=title, abstract, original title, name of substance word, subject heading

word, floating sub-heading word, keyword heading word, organism supplementary concept word, protocol supplementary concept word, rare disease supplementary concept word, unique identifier, synonyms] (0)

16 (knee* adj5 Schatzker*).mp. [mp=title, abstract, original title, name of substance word, subject heading word,

floating sub-heading word, keyword heading word, organism supplementary concept word, protocol supplementary concept word, rare disease supplementary concept word, unique identifier, synonyms] (6)

17 11 or 12 or 13 or 14 or 15 or 16 (18724)

18 10 or 17 (24556)

Annotation: Tibia/knee_ fractures

19 (proximal or upper or plateau or intra-articular or intraarticular or periarticular or peri-articular).mp.

[mp=title, abstract, original title, name of substance word, subject heading word, floating sub-heading word, keyword heading word, organism supplementary concept word, protocol supplementary concept word, rare disease supplementary concept word, unique identifier, synonyms] (593725)

20 18 and 19 (5153)

21 Arthroplasty, Replacement, Knee/ (22515)

22 Arthroplasty, replacement/ (5923)

23 Arthroplasty/ (8560)

24 replacement.mp. (294621)

25 TEP.mp. (2824)

26 arthroplasty.mp. (81565)

27 22 or 23 or 24 or 25 or 26 (322400)

28 20 and 27 (475)

29 8 and 19 and 21 (291)

30 28 or 29 (540)

31 exp Animals/ (22758838)

32 Humans/ (18115821)

33 31 not 32 (4643017)

34 30 not 33 (535)
